# Consequences of microbial diversity in forest nitrogen cycling: diverse ammonifiers and specialized ammonia oxidizers

**DOI:** 10.1038/s41396-019-0500-2

**Published:** 2019-09-03

**Authors:** Kazuo Isobe, Yuta Ise, Hiroyu Kato, Tomoki Oda, Christian E. Vincenot, Keisuke Koba, Ryunosuke Tateno, Keishi Senoo, Nobuhito Ohte

**Affiliations:** 1 Graduate School of Agricultural and Life Sciences, The University of Tokyo, Tokyo, Japan; 2 Graduate School of Informatics, Kyoto University, Kyoto, Japan; 3 Center for Ecological Research, Kyoto University, Kyoto, Japan; 4 Field Science Education and Research Center, Kyoto University, Kyoto, Japan; 5 Collaborative Research Institute for Innovative Microbiology, The University of Tokyo, Tokyo, Japan

**Keywords:** Microbial ecology, Forest ecology, Biogeochemistry

## Abstract

We tested the ecosystem functions of microbial diversity with a focus on ammonification (involving diverse microbial taxa) and nitrification (involving only specialized microbial taxa) in forest nitrogen cycling. This study was conducted on a forest slope, in which the soil environment and plant growth gradually changed. We measured the gross and net rates of ammonification and nitrification, the abundance of predicted ammonifiers and nitrifiers, and their community compositions in the soils. The abundance of predicted ammonifiers did not change along the soil environmental gradient, leading to no significant change in the gross ammonification rate. On the other hand,  the abundance of nitrifiers and the gross nitrification rate gradually changed. These accordingly determined the spatial distribution of net accumulation of ammonium and nitrate available to plants. The community composition of predicted ammonifiers gradually changed along the slope, implying that diverse ammonifiers were more likely to include taxa that were acclimated to the soil environment and performed ammonification at different slope locations than specialized nitrifiers. Our findings suggest that the abundance of ammonifiers and nitrifiers directly affects the corresponding nitrogen transformation rates, and that their diversity affects the stability of the rates against environmental changes. This study highlights the role of microbial diversity in biogeochemical processes under changing environments and plant growth.

## Introduction

The role of microbial diversity in ecosystem functions has been a focus of debate [1–3]. However, most ecologists typically assume that a diverse community has greater functional redundancy than a community compromising specialized taxa [4, 5]. This assumption has been developed by theoretical or empirical studies of macroorganisms, such as plants and animals [6–8]. Microbial ecological studies, testing whether more diverse microbial communities are more functionally redundant, that is, whether changes in community composition less likely correspond with changes in functional rates [3], can support this assumption [9–11]. However, such studies have been mostly limited to small-scale experiments (e.g., soil microcosm experiments in laboratories) with the microbial diversity being experimentally manipulated.

Microbes dominate the biogeochemistry of ecosystems by virtue of their numbers and metabolic capabilities [12, 13]. Thus, we could expect that the greater functional redundancy of diverse microbial communities, if true, affects biogeochemical cycling in an ecosystem. Based on the assumption of microbial functional redundancy, Schimel [14] proposed that, because nitrogen (N) cycling and carbon (C) cycling comprise broad (involving diverse microbial taxa) and narrow (involving only specialized microbial taxa) processes, the broad processes may be more stable against environmental change than the narrow ones. However, this has rarely been tested at the terrestrial ecosystem level [15, 16].

Nitrogen supply to plants via microbial metabolic functions can limit plant productivity in temperate forests [17]. Ammonium produced by ammonifying microbes and NO_3_^−^ produced by nitrifying microbes are the main N species in the soil available to plants. Ammonifying microbes, which generate NH_4_^+^ from organic N monomers, are considered more phylogenetically diverse than nitrifying microbes, which oxide NH_3_/NH_4_^+^ to NO_2_^−^/NO_3_^−^ [18–20]. Moreover, we could expect that most soil microbes regardless of their phylogeny participate in ammonification. Taking up organic N monomers is an essential metabolic process for microbes, and deaminases and deamidases that generate the precursors of NH_4_^+^ are active inside living microbes [18–20]. Conversely, nitrifying microbes, in particular, autotrophic and aerobic NH_3_-oxidizing microbes that mediate the rate-limiting step of nitrification, are phylogenetically and metabolically limited within the phyla Proteobacteria (NH_3_-oxidizing bacteria), Thaumarchaeota (NH_3_-oxidizing archaea), and potentially Nitrospirae (recently found comammox bacteria) [21, 22]. Thus, if a more diverse microbial community has greater functional redundancy, we could expect that ammonification would be more stable against environmental change than nitrification, and such differences could affect the availability of soil N to plants.

The abundance of microbes possessing specialized functions is frequently correlated with the rates of the ecosystem processes that they perform, such as carbon degradation [23], NH_4_^+^ production [24], nitrification [25–27], and denitrification [28]. In contrast, many studies that have assessed the overall microbial community composition (or diversity) and ecosystem process rates have not reported a direct link between them [29]. A recent meta-analysis found that the inclusion of community composition as a predictor of C- and N-cycling process rates improved predictive power in only 29% of considered studies [30]. Thus, we hypothesized that the abundance of microbes possessing specialized functions directly affects the corresponding ecosystem process rates, and that their diversity affects the stability of the process against environmental changes.

This study aimed to test the aforementioned hypotheses with a focus on ammonification and nitrification, which influence plant productivity [17], in a forest. We conducted this study on a steep forest slope wherein soil hydrological and chemical properties and plant productivity gradually change at a small scale. We specifically analyzed the effects of the following variables: (1) the soil environmental gradient on gross ammonification and nitrification rates with the change in the abundance of ammonifiers and nitrifiers, (2) differences in the phylogenetic diversity of ammonifiers and nitrifiers on the stability of gross ammonification and nitrification rates, and (3) the identified relationships (i.e., 1 and 2) on ammonium and nitrate contents available to plants (i.e., net ammonification and nitrification rates) in the soils.

## Materials and methods

### Study site

The study was conducted in a planted coniferous forest in the Fukuroyamasawa Experimental Watershed located in The University Forest in Chiba, the University of Tokyo, Japan (35° 12′ N, 140° 06′ E). The forest is located in a temperate climatic region and a mountainous area at an elevation of 124–227 MASL. The mean annual precipitation and air temperature (1994–2003) were 2230 mm and 14 °C, respectively [31].

We established a 10 × 100-m^2^ study plot in September 2013 on a steep slope (ca. 30°) from the ridge to the valley in a small-scaled (1.09 ha) watershed (Fig. S1). The evergreen coniferous tree species *Cryptomeria japonica* was planted from 1928 to 1930. The growth rate of the tree species increases down the slope. The height was 19.2 ± 4.2 m (*n* = 44) around the upper end of the slope and 26.9 ± 5.0 m (*n* = 129) around the lower end of the slope (100 m from the ridge). The diameter at breast height was 30.8 ± 10.5 cm around the upper end and 37.7 ± 13.0 cm around the lower end [31]. The volumetric soil water content at a 10-cm depth at the ridge and valley was monitored over time during the actively growing season of trees [32] from May 11, 2014 to August 4, 2014 using time domain reflectometry. The details are described in the Supplementary Methods.

### Soil sampling and chemical analysis

Soil sampling was conducted twice (September 2013 and August 2014). We sampled ~200 g of soil from the surface 0–10 cm deep in the mineral layer (A horizon). In September 2013, we sampled 55 soil samples at 11 points (every 10 m from the ridge to the valley) with five replicates at each point by sampling every 2 m from the left to right side (Fig. S1). In August 2014, we sampled ten soil samples at the ridge and valley (100 m from the ridge) with five replicates. The soils were sieved through a 2-mm mesh. Soil sampled in September 2013 was used to survey the spatial variations of N biogeochemical properties, microbial abundances, and microbial community compositions. Soil sampled in August 2014 was used in the water manipulation experiment.

The water content, pH, NH_4_^+^ content, NO_3_^−^ content, and dissolved organic nitrogen (DON) and carbon (DOC) contents of soils were measured, the details of which are described in the Supplementary Methods.

### Measurement of the gross and net ammonification and nitrification rates

We determined the gross and net rates of ammonification and nitrification in the soils. The gross rates are the actual rates, and thus are reflected directly by the microbial reactions of ammonification and nitrification [33]. The net rates (or the net accumulation of NH_4_^+^ and NO_3_^−^ in the absence of plant roots) are the excess N after the microbial consumption of produced NH_4_^+^ and NO_3_^−^, and thus are thought to provide a good index of N availability to plants [33].

The gross ammonification and nitrification rates in soil samples were determined during 24 h of incubation using the isotope dilution method [33]. The net ammonification and nitrification rates were calculated as the concentration changes in soil NH_4_^+^ and NO_3_^−^, respectively, without ^15^N addition during the 28-day incubation. The measurement followed previously described methods [34, 35], the details of which are described in the Supplementary Methods.

### Quantification of 16S rRNA gene, bacterial and archaeal *amoA*, and bacterial *nirK*

We hypothesized that we could consider almost all bacteria to be ammonifiers because NH_4_^+^ production can occur via the assimilation of small organic N compounds that all bacteria can facilitate [19, 20]. We analyzed ammonia-oxidizing bacteria and archaea as nitrifiers, which are responsible for ammonia oxidation, the rate-limiting step of nitrification [36], and nitrite-reducing bacteria as denitrifiers, which are responsible for the indispensable step of denitrification, namely, the reduction of soluble nitrite to gaseous nitric oxide [37].

Microbial DNA was extracted from the soils and purified as described previously [27]. Abundances of the bacterial 16S rRNA gene, bacterial and archaeal ammonia monooxygenase genes (*amoA*), and bacterial copper-containing reductase gene (*nirK*) were quantified by qPCR to estimate the abundances of total bacteria, bacterial and archaeal nitrifiers, and bacterial denitrifiers, respectively. The quantification followed the methods described by Isobe et al. [38], and the details are described in the Supplementary Methods.

### Sequence analysis of 16S rRNA gene

Bacterial 16S rRNA genes in soils were PCR-amplified using the primers 515f-806r. These primers were identical to those used in the qPCR assay, except for the addition of appropriate Illumina adapters and 12-bp barcodes for multiplex sequencing on the Illumina platform [39]. PCR amplification, sequencing, and sequence analysis followed the methods described by Isobe et al. [38], the details of which are described in the Supplementary Methods. The UPARSE pipeline [40] was used to merge the de-multiplexed sequences, conduct quality filtering, and cluster sequences into operational taxonomic units (OTUs) at >97% sequence similarity. The number of sequences per sample was rarefied to 17,800 via random sampling within the QIIME pipeline [41]. The OTUs’ sequences were aligned with PyNAST, and a phylogenetic tree was constructed with FastTree after the aligned sequences were filtered with the default lanemask file within QIIME.

PCR primers for quantifying the abundances of microbial genes responsible for ammonification have not been developed, unlike *amoA* in nitrification and *nirK* and *nirS* in denitrification. Thus, to estimate the abundance and composition of these microbial genes, we used Phylogenetic Investigation of Communities by Reconstruction of Unobserved States (PICRUSt) [42], which was designed to computationally infer metagenome functional contents from 16S rRNA gene sequences. The detailed procedures are described in the Supplementary Methods. We predicted the occurrence of the gene necessary for extracellular enzymes to degrade plant litter or dead microbes, namely, *N*-acetylglucosaminidase (EC 3.2.1.52). We also predicted the occurrence of the genes necessary for intracellular enzymes for NH_3_ production, namely, arginase (EC 3.5.3.1) and urease (EC 3.5.1.5). The reactions of these enzymes can be considered as rate-limiting steps of ammonification, and thus are frequently measured to estimate microbial ammonification activity [43]. Furthermore, the presence of *N*-acetylglucosaminidase gene is known to be phylogenetically conserved [44], and thus 16S rRNA gene-based phylogeny may enable estimation of the production capability at relatively high probability.

### Clone library analysis of bacterial and archaeal *amoA*

To estimate the community composition of nitrifiers, we filtered the ammonia oxidizers’ OTUs based on their 16S rRNA gene-based taxonomic information. Accordingly, we found that our sequence data possessed the OTUs of the known archaeal or bacterial ammonia oxidizers. However, those OTUs were not detected in the upper slope. Therefore, we performed small-scaled clone library analysis of their *amoA* sequences and compared the community composition of nitrifiers in the upper and lower slopes. We used microbial DNA extracted from one soil sample each from the ridge and valley. The bacterial and archaeal *amoA* sequences were amplified with the primers used in the qPCR assay, which obtained 37 and 40 bacterial *amoA* clones and 48 and 47 archaeal *amoA* clones from the ridge and valley, respectively. Then, we obtained three and four bacterial *amoA* OTUs and six and eleven archaeal *amoA* OTUs from the ridge and valley, respectively. PCR amplification, Sanger sequencing, OTU clustering (>97% sequence similarity), and phylogenetic analysis followed the methods described by Isobe et al. [26], details of which are described in the Supplementary Methods.

### Water manipulation experiment

We expected from the results of the survey of the spatial variations that soil water content strongly affects the abundance of nitrifiers and the nitrification rate in soil, but does not affect the bacterial community composition. Thus, we monitored their abundance and NO_3_^−^ content in soil with/without experimental water addition. We used the three soil sets: soils taken at the ridge (dry soils, 23% soil water content on average), soils taken at the valley (wet soils, 43%), and soils taken at the ridge with water added to the same level as that in soils at the valley (wet soils, 43%). Twenty grams of soil were dispensed into the glass vessels covered with aluminum foil to minimize water evaporation and incubated for 0, 14, 28, 42, or 84 days at 20 °C in the dark. Water loss was replenished at these days. Then, the soil nitrate content was measured. The bacterial and archaeal *amoA* and 16S rRNA gene abundances were measured at 0, 42, and 84 days. The 16S rRNA gene was sequenced at 0 and 84 days and rarefied at 13,000 sequences. All analyses including gene quantification and the sequence analysis of 16S rRNA gene were conducted as described above.

### Statistical analysis

The details are described in the Supplementary Methods. Briefly, the relationships of the slope position with the measured variables were tested via correlation analysis. Structural equation modeling (SEM [45]) was used to determine the effects of soil environmental gradient on ammonification and nitrification rates by affecting the abundance of ammonifiers and nitrifiers. We tested the simple hypothetical relationship involving the gross ammonification and nitrification rates being determined by the abundance of ammonifiers and nitrifiers as well as the substrate for the processes, and the abundance of ammonifiers and nitrifiers being determined by soil environmental properties (i.e., soil pH and water content) as well as the substrate. Spatial change in the community composition was visualized via a nonmetric multidimensional scaling (NMDS) ordination with Bray–Curtis similarities. Then, the community composition was statistically compared among the slope positions via a permutational multivariate analysis of variance (PERMANOVA) test. The Bray–Curtis similarity between two communities was also statistically compared with the geographic distance between the communities via a Mantel test. The soil environmental variables that strongly correlated with the compositional similarity were tested for significance based on a permutation test.

## Results

### Soil environmental properties

**Fig. 1:**
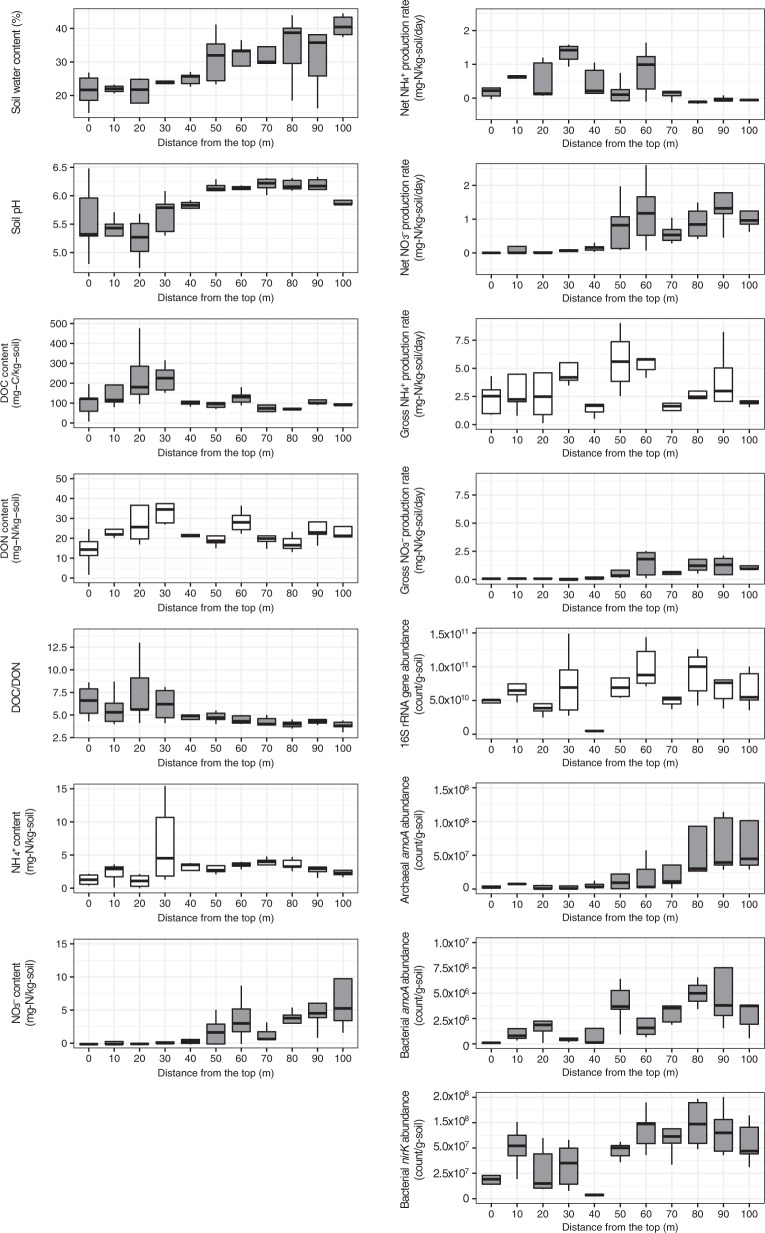
Soil environmental properties, nitrogen transformation rates, and microbial gene abundances along the forest slope. The variables exhibiting significant relationships with the location on the forest slope are displayed in gray (*p* < 0.05)

Soil water content increased down the slope from 21.4% at the ridge to 40.8% at the valley (*R* = 0.71, *p* < 0.001, Fig. 1). The soils at the ridge were constantly drier than those at the valley over ~3 months of observation (Fig. S1). Soil pH also increased down the slope from 5.2–5.6 at the ridge to 5.9–6.2 at the valley (*R* = 0.62, *p* < 0.001, Fig. 1). Soil water content increased more linearly than soil pH.

### N transformations

Soil NO_3_^−^ content increased down the slope (*R* = 0.56, *p* < 0.001, Fig. 1), whereas no consistent increase or decrease was noted for soil NH_4_^+^ content (*R* = 0.13, *p* = 0.34, Fig. 1). Soil DON content did not exhibit a consistent spatial change (*R* = −0.07, *p* = 0.60, Fig. 1), but soil DOC content (*R* = −0.31, *p* = 0.02, Fig. 1) and DOC/DON (*R* = −0.53, *p* < 0.001, Fig. 1) decreased down the slope.

Net ammonification and nitrification rates along the slope were within the range of variation in the rates of forests in Japan (−0.16 to 0.98 and 0.0 to 3.00 mg N kg^–1^ d^–1^, *N* = 38 [46]). Gross ammonification and nitrification rates along the slope were also within the range of variation in the rates of forests in Japan (0.82 to 15.83 and 0.04 to 3.78 mg N kg^–1^ d^–1^, *N* = 38 [46]). The net ammonification rate decreased down the slope (*R* = −0.43, *p* < 0.001, Fig. 1), exhibiting positive values for the upper slope (0 to 40 m from the top) and negative values for the lower slope (80 to 100 m from the top). Conversely, the net nitrification rate increased down the slope (*R* = 0.61, *p* < 0.001, Fig. 1), being positive for the lower slope (40–100 m from the top). The gross ammonification rate did not display a consistent spatial change (*R* = −0.10, *p* = 0.48, Fig. 1), but the gross nitrification rate increased down the slope (*R* = 0.57, *p* < 0.001, Fig. 1).

### Microbial abundances

The abundance of the 16S rRNA gene did not exhibit a consistent spatial change (*R* = 0.48, *p* = 0.08, Fig. 1), but the abundances of both archaeal and bacterial *amoA* increased down the slope (*R* = 0.52 for archaeal *amoA*, *R* = 0.48 for bacterial *amoA*, *p* < 0.001 for both, Fig. 1). Archaeal *amoA* was more abundant than bacterial *amoA* at all locations along the slope. The abundance of bacterial *nirK* also increased down the slope (R = 0.54, *p* < 0.001, Fig. 1). We assumed that most bacteria can participate in ammonification, and observed a weak but positive correlation between the abundance of 16S rRNA gene and the gross ammonification rate (*R* = 0.34, *p* = 0.01). We also observed a positive correlation between the abundance of archaeal *amoA* and gross nitrification rate (*R* = 0.45, *p* < 0.001), but not between the abundance of bacterial *amoA* and gross nitrification rate (*R* = 0.20, *p* = 0.16).

### Relationships among soil environmental properties, microbial abundances, and N transformations

**Fig. 2:**
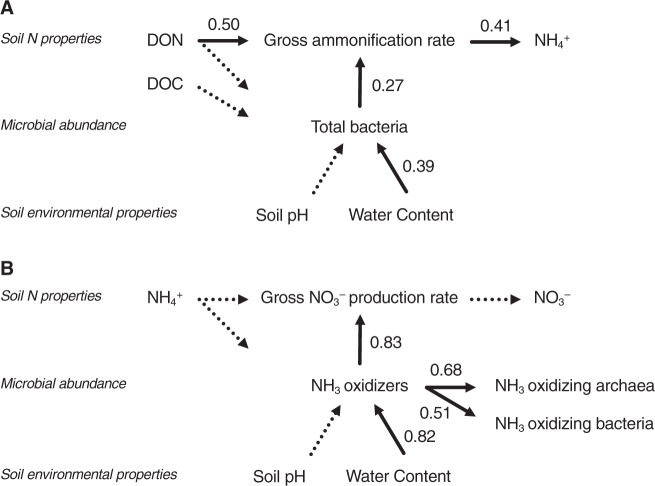
Hierarchical relationship among soil environmental properties, microbial abundances, and nitrogen transformations. The path diagrams represent the full and final models used to describe the relationship between the gross ammonification rate (**a**) and gross nitrification rate (**b**). The numbers associated with the arrows between two variables are the partial regression coefficients derived from multiple regressions. The solid and dashed arrows were significant (*p* < 0.05) and insignificant in the final model, respectively

We examined the relationships among the soil environmental properties, microbial abundances, and N transformations by using the SEM. SEM revealed that the gross ammonification rate was most strongly affected by the soil DON content (effect size = 0.50, *p* < 0.01, Fig. 2), followed by the abundance of total bacteria (effect size = 0.27, *p* < 0.01, Fig. 2). The abundance of total bacteria was affected by the soil water content (effect size = 0.39, *p* < 0.01, Fig. 2), but not by the substrate/energy source (soil DOC or DON content). The gross nitrification rate was affected solely by the abundance of nitrifiers (effect size = 0.83, *p* < 0.01, Fig. 2). The abundance of nitrifiers was strongly affected by the soil water content (effect size = 0.82, *p* < 0.01, Fig. 2), but not by the substrate/energy source (soil NH_4_^+^ content).

### Microbial community composition

**Fig. 3:**
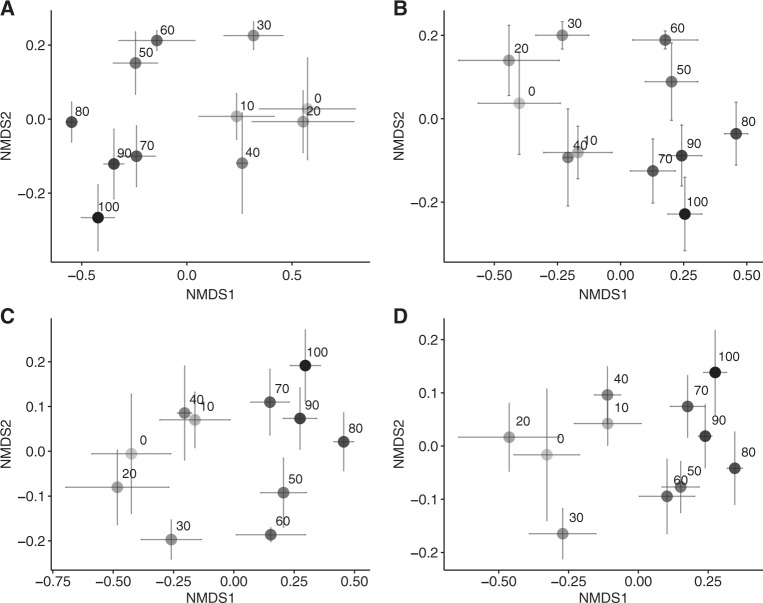
Nonmetric multidimensional scaling ordination showing the change in the composition of total bacterial community (**a**) and operational taxonomic units predicted to possess the genes for *N*-acetylglucosaminidase (**b**), arginase (**c**), and urease (**d**) along the forest slope. The numbers are the distances from the ridge (m)

From the 16S rRNA gene sequences rarefied to 17,800 sequences, 10,651 OTUs were obtained. The microbial community was dominated by the phyla Proteobacteria and Acidobacteria, followed by Actinobacteria, Verrucomicrobia, and Planctomycetes (Fig. S2). The alpha diversity represented by the number and Shannon diversity index of OTUs in 17,800 sequences increased down the slope (Fig. S3). The overall community composition shifted along the slope (PERMANOVA: *R* = 0.47, *p* < 0.001, Fig. 3) and community compositions at more distant locations became more different (Mantel test for Bray–Curtis similarity: *R* = –0.428, *p* < 0.001, Fig. S4). When exploring the soil environmental variables most strongly correlated with the compositional similarity, soil pH was the largest factor influencing the composition (*R* = 0.64, *p* < 0.001), followed by soil NH_4_^+^ content (*R* = 0.55, *p* = 0.001) and soil DOC/DON (*R* = 0.39, *p* = 0.04).

PICRUSt estimated counts of the genes based on the 17,800 rarefied 16S rRNA gene sequences, tagged with KEGG Orthology. We then extracted the counts of K01207 for N-acetylglucosaminidase, K01476 for arginase, and K01427 for urease (subunit alpha). The Nearest Sequenced Taxon Index score for our PICRUSt analysis was 0.21 ± 0.02 sd (*N* = 55), slightly higher than values in the soils (0.17 ± 0.02 sd) described in the paper by Langille et al. [42], the original publication of PICRUSt that showed relatively accurate functional gene predictions.

**Fig. 4:**
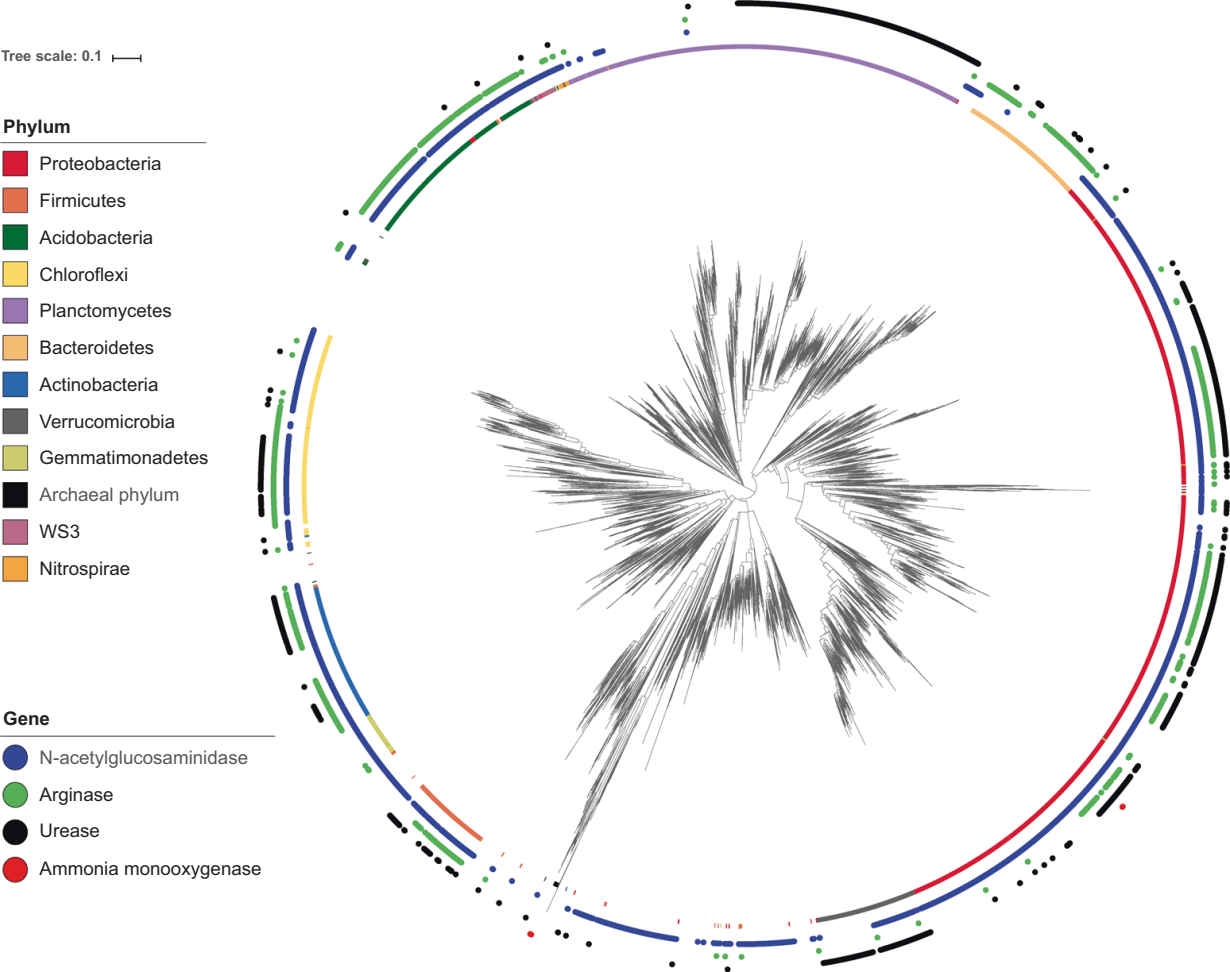
Phylogenetic diversity of operational taxonomic units (OTUs) predicted to possess the genes for *N*-acetylglucosaminidase, arginase, and urease and identified as archaeal and bacterial ammonia oxidizers. These OTUs were mapped onto the phylogenetic tree of all OTUs along the forest slope. The inner ring shows the taxonomy at the phylum level. The phylum with their abundance >1% were illustrated. For a small portion of OTUs, taxonomic assignment did not match tree topology; however, no manual curation was attempted

The OTUs predicted to possess the genes necessary for *N*-acetylglucosaminidase, arginase, and urease production were mapped onto the phylogenetic tree, illustrating that they were widely distributed throughout the different phyla (Fig. 4). The absolute abundance of those OTUs, estimated by the counts of the gene multiplied by the abundance of 16S rRNA gene in the soil, did not display spatial changes along the slope (Fig. S5), but it exhibited a positive correlation with the gross ammonification rate (*R* = 0.31, *p* = 0.02 for *N*-acetylglucosaminidase; *R* = 0.31, *p* = 0.02 for arginase; *R* = 0.36, *p* < 0.01 for urease). The compositions of those OTUs shifted along the slope (PERMANOVA: *R* = 0.47, *p* < 0.001 for *N*-acetylglucosaminidase; *R* = 0.47, *p* < 0.001 for arginase; *R* = 0.48, *p* < 0.001 for urease, Fig. 3; Mantel test for Bray–Curtis similarity: *R* = −0.40, *p* < 0.001 for *N*-acetylglucosaminidase; *R* = −0.40, *p* < 0.001 for arginase; *R* = −0.39, *p* < 0.001 for urease, Fig. S4). The relative abundance of 1159 and 2273 OTUs increased up the slope and down the slope, respectively (linear regression, *p* < 0.1, Table 1), suggesting that they became more dominant in the community in the upper and lower parts, respectively. A substantial proportion of them was predicted to possess the genes necessary for ammonification (Table 1).

**Table 1:** The number of OTUs predicted to possess the genes for ammonification and ammonia oxidation

	Total	*N*-acetylglucosaminidase	Arginase	Urease	Ammonia monooxygenase
Upper slope	1159	774	410	482	0
Lower slope	2273	1407	691	631	5
Nonlinear	7219	4175	1752	1936	3

OTUs were classified into three categories where their relative abundance increased at the upper or lower slope by exhibiting the linear relationships with the location on the forest slope (upper or lower slope) or did not increase significantly at either of upper or lower slope (nonlinear) (*p* < 0.1)

The OTUs identified as ammonia oxidizers based on their 16S rRNA gene-based taxonomy were also mapped onto the phylogenetic tree (Fig. 4). Unlike ammonifiers, these OTUs were located in specific phylogenetic branches. The relative abundance of these OTUs increased down the slope (*R* = 0.70, *p* < 0.001 for archaea; *R* = 0.52, *p* < 0.001 for bacteria, Fig. S6). The OTUs of which relative abundance increased at the upper slope did not possess the genes for ammonia oxidation (Table 1). We could not analyze the change in their community composition because they were not detected along the upper slope (Fig. S6). Therefore, we compared the community composition based on *amoA* sequence clone libraries in the soils at the ridge and valley. The community composition of NH_3_-oxidizing archaea differed between the ridge and valley (Fig. S7). Archaeal *amoA* clones belonging to OTU_1, OTU_2, and OTU_6 were dominant at the ridge, whereas those belonging to OTU_2, OTU_3, OTU_4, and OTU_5 were dominant at the valley. Conversely, the community composition of NH_3_-oxidizing bacteria was similar (Fig. S7). Bacterial *amoA* clones belonging to OTU_1 were dominant at both the ridge and valley. Both archaeal and bacterial *amoA* OTUs that were dominant at the ridge were less abundant than the OTUs that were present at the valley (Fig. S7).

### Microbial responses to water manipulation

**Fig. 5:**
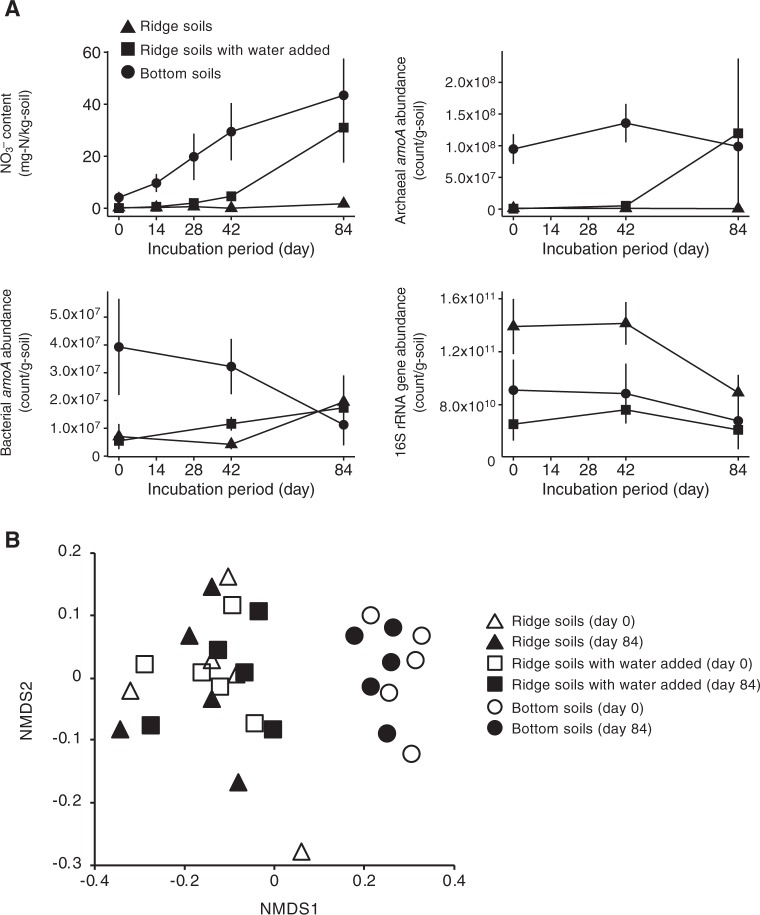
Microbial responses to water manipulation. Nitrate content (**a**) and archaeal (**b**) and bacterial (**c**) *amoA* abundances in three soil sets: soils taken at the ridge (dry soils, 23% soil water content on average), soils taken at the valley (wet soils, 43%), and soils taken at the ridge with water added to the same level as that in soils at the valley (wet soils, 43%). Nonmetric multidimensional scaling ordination showing the change in the composition of the ammonifier (total microbial) community in the three soil sets during the 84-day incubation (**d**)

The NO_3_^−^ content of the soils taken at the valley increased with incubation time, whereas that of the soils taken at the ridge remained low (Fig. 5). The nitrate content of the soils taken at the ridge with water added increased starting on day 28. By day 84, the NO_3_^−^ content of the valley soils or water-manipulated ridge soils was higher than that of the ridge soils (*p* < 0.05). Along with the soil NO_3_^−^ content, the abundance of archaeal *amoA* remained high and low in the soils at the valley and ridge, respectively, and increased from day 28 in the water-manipulated ridge soils (*R* = 0.94, *p* < 0.001 for the correlation between the soil NO_3_^−^ content and abundance of archaeal *amoA*). On day 84, the abundances of archaeal *amoA* of the valley and water-manipulated ridge soils were higher than that of the ridge soils (*p* < 0.05). In contrast, the abundances of bacterial *amoA* and 16S rRNA gene of the valley soils had decreased, and they were comparable with those of the ridge and water-manipulated ridge soils.

From the 16S rRNA gene sequences rarefied to 13,000 sequences, 10,695 OTUs were obtained. The microbial community composition differed between the valley and ridge soils on day 0 (Fig. 5). The community composition of all soil sets did not shift significantly during the 84-day incubation. In particular, on day 84, the community compositions of the ridge soils with and without water manipulation did not differ from each other, but they differed from that of the valley soils (*p* < 0.05).

## Discussion

### Hierarchal relationship of soil environmental properties, microbial abundances, and N transformations

We first analyzed the effects of the soil environmental gradient on gross ammonification and nitrification rates with the change in the abundance of ammonifiers (i.e., total bacteria or OTUs predicted to be responsible for ammonification) and nitrifiers. SEM illustrated the hierarchical relationship of these properties (Fig. 2).

The gross rate measurement showed that NH_4_^+^ was produced at a constant rate at all slope positions but was oxidized to NO_3_^−^ via nitrification only in the lower slope (Fig. 1). This can explain the different N species that accumulated along the upper and lower parts of the slope as illustrated by the net ammonification and nitrification rates; NH_4_^+^ in the upper slope (0–40 m from the top) and NO_3_^−^ in the lower slope (80–100 m from the top) (Fig. 1). These N transformations could be driven by the abundances of ammonifiers and nitrifiers. The abundance of ammonifiers and NH_3_ oxidizing archaea exhibited a similar change along the slope to the gross ammonification and nitrification rates, respectively (Fig. 1 and S5), and showed positive correlations with the corresponding process rates. NH_3_-oxidizing archaea seemed more substantially responsible for nitrification than NH_3_ oxidizing bacteria from the results of correlations (the abundance of archaeal *amoA*, not bacterial *amoA*, correlated with nitrification rate) and the water manipulation experiment discussed below.

The abundances of both ammonifiers and nitrifiers were affected by soil water content, but the abundance of nitrifiers was more sensitive to changes in soil water content (Fig. 2). The higher sensitivity of the abundance of nitrifiers was further confirmed in the water manipulation experiment (Fig. 5). NH_3_-oxidizing archaea in the ridge soils responded positively to water manipulation, which was consistent with the increase in soil NO_3_^−^ content. The enhancement of growth and nitrification activity of soil NH_3_-oxidizing archaea, not NH_3_-oxidizing bacteria, by the increase in water availability was also observed by Bustamante et al. [47].

Thus, we concluded that the gross ammonification and nitrification rates were affected by the abundances of ammonifiers and nitrifiers, respectively. The gross ammonification and nitrification rates were affected differently by the soil water gradient because of the lower sensitivity of abundance of ammonifiers and higher sensitivity of abundance of nitrifiers to the soil water gradient.

### Phylogenetic microbial diversity sustaining the stability of N transformations

We then analyzed the effects of differences in the phylogenetic diversity of ammonifiers and nitrifiers on the stability of ammonification and nitrification rates. Microbial functional redundancy illustrates that even when the composition of a microbial community shifts in the face of environmental changes, the functions of a diverse community are less affected. This occurs because the diverse community is likely to possess taxa that are physiologically well-acclimated to the new environmental conditions and that can complement the functions of affected taxa [3, 15].

Our results suggested that the communities at the upper and lower slopes have different taxa of ammonifiers (Table 1 and Fig. 4), while the abundance of ammonifiers did not exhibit a consistent spatial change along the slope (Fig. 1 and S5). Their community composition was considered to be highly affected by the soil pH. These suggests that different taxa could acclimate to the soil environmental conditions (particularity to soil pH) at different locations along the slope and perform the same function of ammonification.

Conversely, nitrifiers on the slope might not include taxa that are physiologically acclimated to environments of the upper slope. Both archaeal and bacterial *amoA* OTUs that were dominant at the ridge were less abundant than the OTUs that were present at the valley (Fig. S7). Not only that but also NH_3_-oxidizing archaea and bacteria constituted the more minor group in the whole community at the ridge because they were not detected in the 17,800 rarefied sequences (Fig. S6). This suggests that the phylogenetic diversity of NH_3_-oxidizing archaea and bacteria at the ridge were strictly suppressed by the soil environmental conditions of the location. Gubry-Rangin et al. [48] studied the geography of NH_3_-oxidizing archaea and found that NH_3_-oxidizing archaea in low-pH soils were phylogenetically less diverse and constituted the more minor group in the whole community than those in neutral-pH soils. We also found that only one *amoA* OTU was predominant in highly acidified forest soils [26]. Huang et al. [49] studied the archaeal geography and found that archaea in drier soils were phylogenetically less diverse. These findings suggest that NH_3_-oxidizing archaea, the main NH_3_-oxidizers in the soils, are less likely to possess taxa that prefer or adapt to low-pH and/or low-water-content soils in their phylogeny.

Thus, based on the results, we could expect that the larger phylogenetic diversity of ammonifiers facilitates the greater adaptability to new environments and thus the higher stability of the abundance of ammonifiers and ammonification rate against soil environmental changes.

### Functional role of microbial diversity in forest N cycling and plant growth

We discuss the effects of the relationships among soil environmental properties, microbial abundances, microbial diversity, and N transformations on N availability to plants. As discussed above, the phylogenetic diversity of ammonifiers and nitrifiers could result in the spatial distribution of the N species available to plants (i.e., NH_4_^+^ in the upper slope and NO_3_^−^ in the lower slope). Previous studies conducted in other forests also showed similar spatial patterns of NH_4_^+^ and NO_3_^−^ along a slope [24, 50–53]. Further, the same tree species exhibited the different growth rates and N utilization patterns between the upper and lower slopes; the trees along the lower slope grew faster by utilizing NO_3_^−^, which is easily diffused in the soil matrix compared with NH_4_^+^, and abundant water whereas the trees along the upper slope were more likely to distribute their biomass to the belowground sections to take up NH_4_^+^ and scarce water [51, 54, 55]. The tree species on the slope in our study site, namely, *Cryptomeria japonica*, also grew faster along the lower slope (see the Materials and methods section). This could be attributable to the higher availability of NO_3_^−^ and water in the lower slope. The close relationship of spatial distributions of N availability and plant productivity in forest has been also reported in some studies [56, 57].

Based on the observations in previous studies and this work, we suggest that the diversity and spatial distribution of ammonifiers and nitrifiers could affect plant growth through the supply of N available to plants.

### Testing the functional role of microbial diversity in the field

This study suggests that the response of microbial abundance that drives N cycling to environmental change can be affected by the phylogenetic diversity, which finally affects the productivity of plants in forests. The greater functional redundancy that we observed in this study was assumed to be an important function of microbial diversity for biogeochemical cycling [14], but this has rarely been tested in the field.

First, we have to distinguish the contributions of microbes and substrate supply to the corresponding ecosystem process. When comparing functional redundancy of functionally distinct microbial groups, the substrate supply by the precursive reaction can overshadow the difference in functional redundancy and thus needs to be carefully considered [58, 59]. For example, the denitrifiers’ *nirK* abundance increased down the slope (Fig. 1), implying that the denitrification rate may increase down the slope. However, we could not consider the functional redundancy of denitrifiers that is more phylogenetically diverse than nitrifiers [37] as low as that of nitrifiers, because the abundance of denitrifiers could be limited by the substrate supply (NO_3_^−^ production by nitrifiers). Recent studies found that substrate availability could be more important than the denitrifiers’ abundance for denitrification [30] and could affect the ﻿microbial diversity–function relationship of denitrifiers [60].

Second, we have to distinguish the contribution of microbial abundance and community composition (or diversity). Conversely, studies on microbial functional redundancy likely focused disproportionately on community composition (or diversity), which is less directly linked to the ecosystem process rates [29]. Further, microbial abundance and community composition may be regulated dominantly by different environmental factors. At our site, the community composition seemed affected strongly by soil pH, whereas the abundance was affected more strongly by soil water content, as expected based on the results of correlations and the water manipulation experiment where the water input did not induce a compositional shift (Fig. 5). Recent studies show that bacterial preference for soil pH is more deeply conserved in their phylogeny than that for soil water content [61, 62], suggesting that change in soil pH alters the community composition more drastically than the change in soil water content. Bacterial biogeographic studies also show that soil pH could shape the community composition more dominantly than soil moisture (e.g. refs. [63–65]). On the other hand, soil water content might affect their abundance by controlling substrate supply to microbes by altering the solvability and diffusion rate of the substrates in soil matrix, as precisely examined in Stark and Firestone [66] with NH_3_ oxidizers in soil.

Previous studies investigating the microbial functional redundancy or microbial diversity–function relationship attempted to overcome these difficulties by manipulating the microbial diversity and abundance and adding the substrate [67]. These studies support the assumption that broad functions such as respiration and biomass production often seem more resistant to changes in community composition or diversity than narrow functions such as denitrification and degradation of specific compounds [9–11, 60, 68], with some exceptions [69]. However, these studies were limited mostly to laboratory incubation and short-term response of manipulated microbial community to the substrate input, which does not always reflect conditions in the field. We shed light on the microbial functional redundancy in the field and discussed its importance in ecosystem N cycling by focusing on functionally distinct microbial groups with contrasting ranges of phylogenetic diversity. We overcame the difficulties above by taking advantage of the distinct characteristics of the study site: the substrate for ammonifiers (soil DON content) and nitrifiers (soil NH_4_^+^ content) did not seem to change along the slope (Fig. 1); and the chemical quality of litter may also not change significantly in the small-scale plot where a single-planted tree species was distributed. Further, we used the steep slope of forests where the soil environmental properties and tree growth changed gradually, which allowed us to estimate the relationship between microbial abundance or community composition and soil environmental properties by simple correlations with the support of a manipulation experiment.

## Conclusion and implications for future research

We demonstrated that the abundance of microbes possessing specialized functions directly affects the corresponding ecosystem process rates and that their diversity affects the stability of the process against environmental changes. By focusing on ammonifiers and nitrifiers in a forest, we further demonstrated that microbial diversity can contribute not only to ecosystem process rates but also to plant productivity.

The microbial functional redundancy may also be important when considering the ecosystem response to a changing environment. The effects of environmental change may be more likely to be evident in the microbial ecosystem processes that are performed by the less diverse or specified microbial taxa. In line with this, nitrification is known as a process sensitive to environmental change because of the large fluctuation of nitrifiers’ abundance upon environmental disturbances [70]. We have also shown that ammonia-oxidizing archaea were sensitive to the ecological phenomena behind the forest N saturation although ammonifying activity was stable, which accordingly caused high NO_3_^–^ leaching from the forest [26]. Therefore, we suggest that microbial functional diversity is critical to improving terrestrial nitrogen models and predicting ecosystem responses to environmental change.

We should note that this study has limitations and thus there is still room for improvement to clearly verify the ecosystem role of microbial diversity. For example, we did not study the fungal activity. Fungi may contribute to ammonification although their contribution is difficult to measure [71]. Further, arbuscular mycorrhizal fungi form symbiotic relationships with *Cryptomeria japonica* and may transfer N, although the trees may take up most available N species in soils regardless of the presence of symbiotic mycorrhiza [72]. In addition, we generally measure ammonification in the form of ammonium production as a single process; however, it is actually the sum of multiple distinct physiological processes, in which multiple enzymes are involved. The relationship between taxonomy and production capabilities for those enzymes is obscure, which hinders the accurate estimation of PICRUSt. These limitations of this study need to be addressed in future studies and we need more study cases to highlight the contributions of microbial diversity and functional redundancy to biogeochemical cycling in an ecosystem.

### Data accessibility

Raw sequence reads from Illumina Miseq were submitted to the DNA Data Bank of Japan (DDBJ) under accession number DRA007963. AmoA clone sequences were submitted to DDBJ under accession numbers LC492980–LC492999. Our analysis of the sequence dataset is available at https://github.com/kazuo-isobe/forest_slope/.

## Supplementary information

Supplementary Information
