# Supplementary material for: Consequences of microbial diversity in forest nitrogen cycling: diverse ammonifiers and specialized ammonia oxidizers
Source: ISME J. 2019 Sep 3;14(1):12–25. doi: 10.1038/s41396-019-0500-2 (PMC6908637; doi:10.1038/s41396-019-0500-2)
Supplement: Supplementary file 1 — Supplementary Information [file 41396_2019_500_MOESM1_ESM.pdf]

Supplementary figures

Title: Consequences of microbial diversity in forest nitrogen cycling: Diverse ammonifiers and specialized ammonia oxidizers

Authors:
Kazuo Isobe[1*], Yuta Ise[1], Hiroyu Kato[1], Tomoki Oda[1], Christian E. Vincenot[2], Keisuke Koba[3], Ryunosuke Tateno[4], Keishi Senoo[1], Nobuhito Ohte[2]

Affiliations:
1 Graduate School of Agricultural and Life Sciences, The University of Tokyo, Tokyo, Japan
2 Graduate School of Informatics, Kyoto University, Kyoto, Japan
3 Center for Ecological Research, Kyoto University, Kyoto, Japan
4 Field Science Education and Research Center, Kyoto University, Kyoto, Japan

Corresponding author:
*Kazuo Isobe
akisobe@mail.ecc.u-tokyo.ac.jp
1-1-1 Yayoi Bunkyo-ku Tokyo, 113-8657 Japan

Figure S1. Geography of the study sites (A) and volumetric soil water content at a 10-cm depth at the ridge and valley (100 m from the ridge) monitored over time from May 11, 2014, to August 4, 2014. Frequency on the Y-axis represents the frequency (the number of days) of given soil water contents during the whole monitoring period.

Figure S2. The taxonomic composition at the phylum level of the soil microbial communities along the forest slope. The phylum with their abundance < 1% were classified into "other phylum".

Figure S3. The number and Shannon diversity index of OTUs in the rarefied 17,800 sequences along the forest slope.

Figure S4. Bray-Curtis similarity between two communities plotted against the geographic distance between the communities for the composition of the ammonifier (total microbial) community (A) or operational taxonomic units predicted to possess the genes for *N*-acetylglucosaminidase (B), arginase (C), and urease (D). Only the distance along the slope was considered.

Figure S5. The relative abundance of operational taxonomic units (OTUs) within a community that was predicted to possess the genes for *N*-acetylglucosaminidase, arginase, urease along the forest slope (A). The estimated abundance of those OTUs in a gram of soil (B).

Figure S6. The relative abundance of operational taxonomic units that were identified as archaeal or bacterial ammonia oxidizers based on their 16S rRNA gene-based taxonomy at the forest slope.

Figure S7. Phylogenetic distributions and estimated abundance of the operational taxonomic units of archaeal (A) and bacterial *amoA* (B) in soils at the ridge and valley (100 m from the ridge).

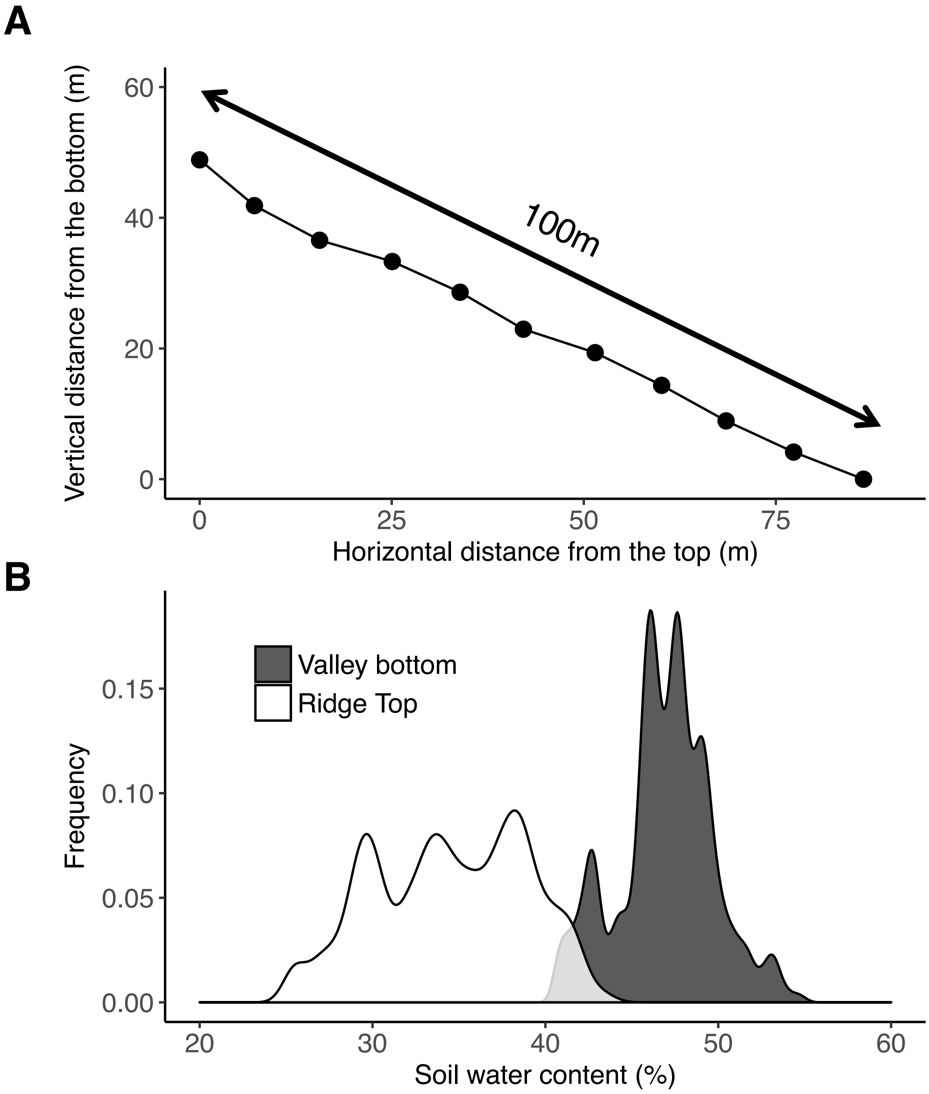

Figure S1 Isobe et al.

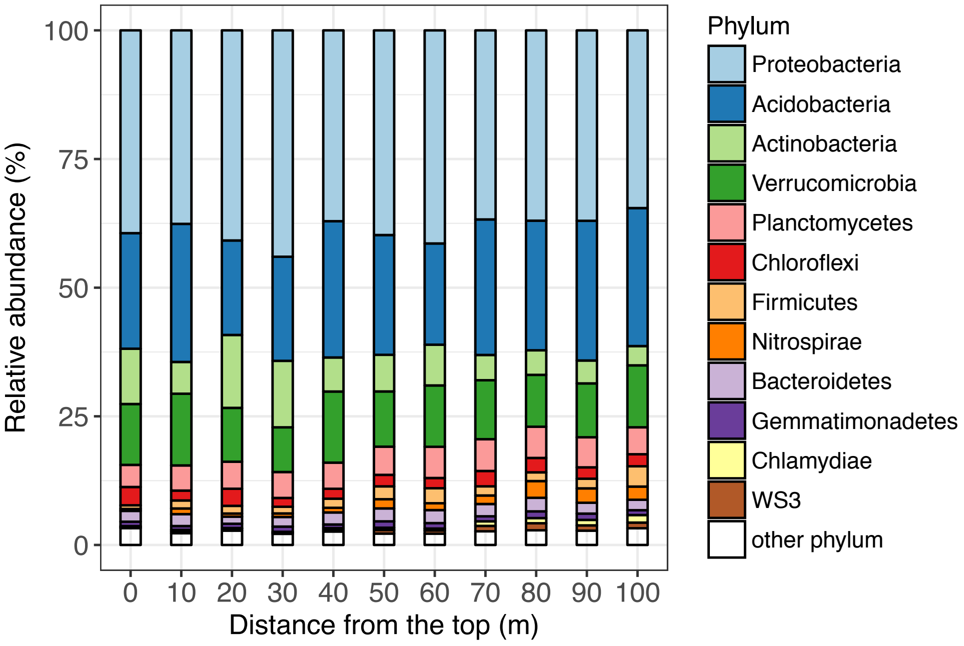

Figure S2 Isobe et al.

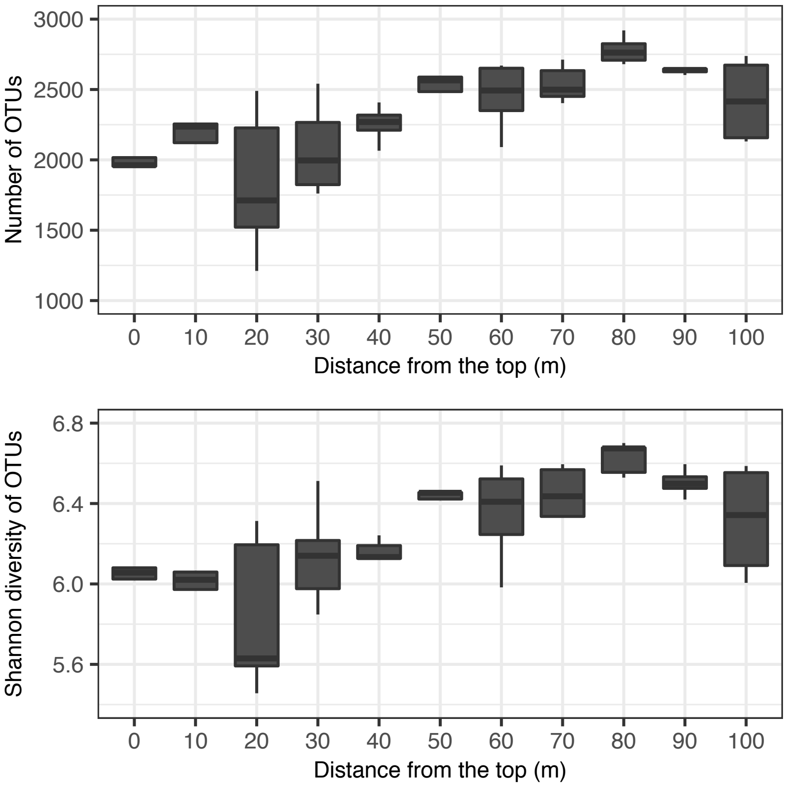

Figure S3 Isobe et al.

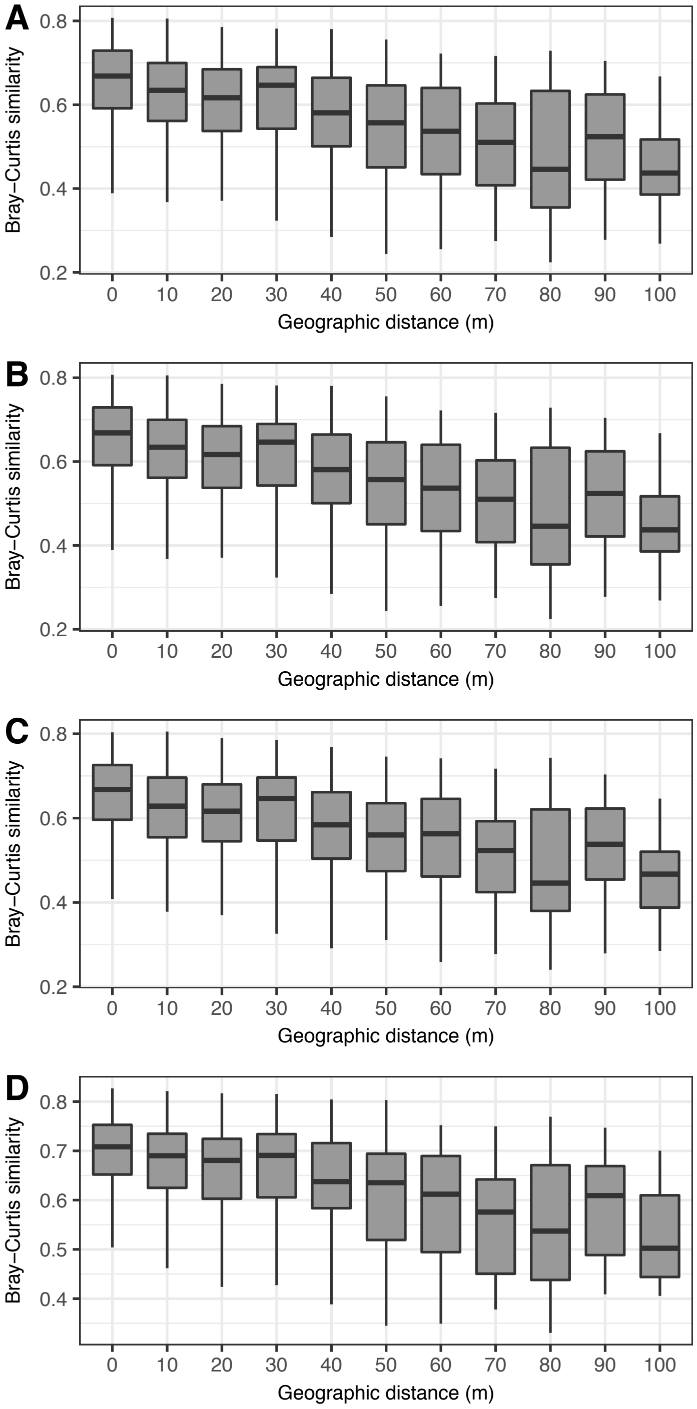

Figure S4 Isobe et al.

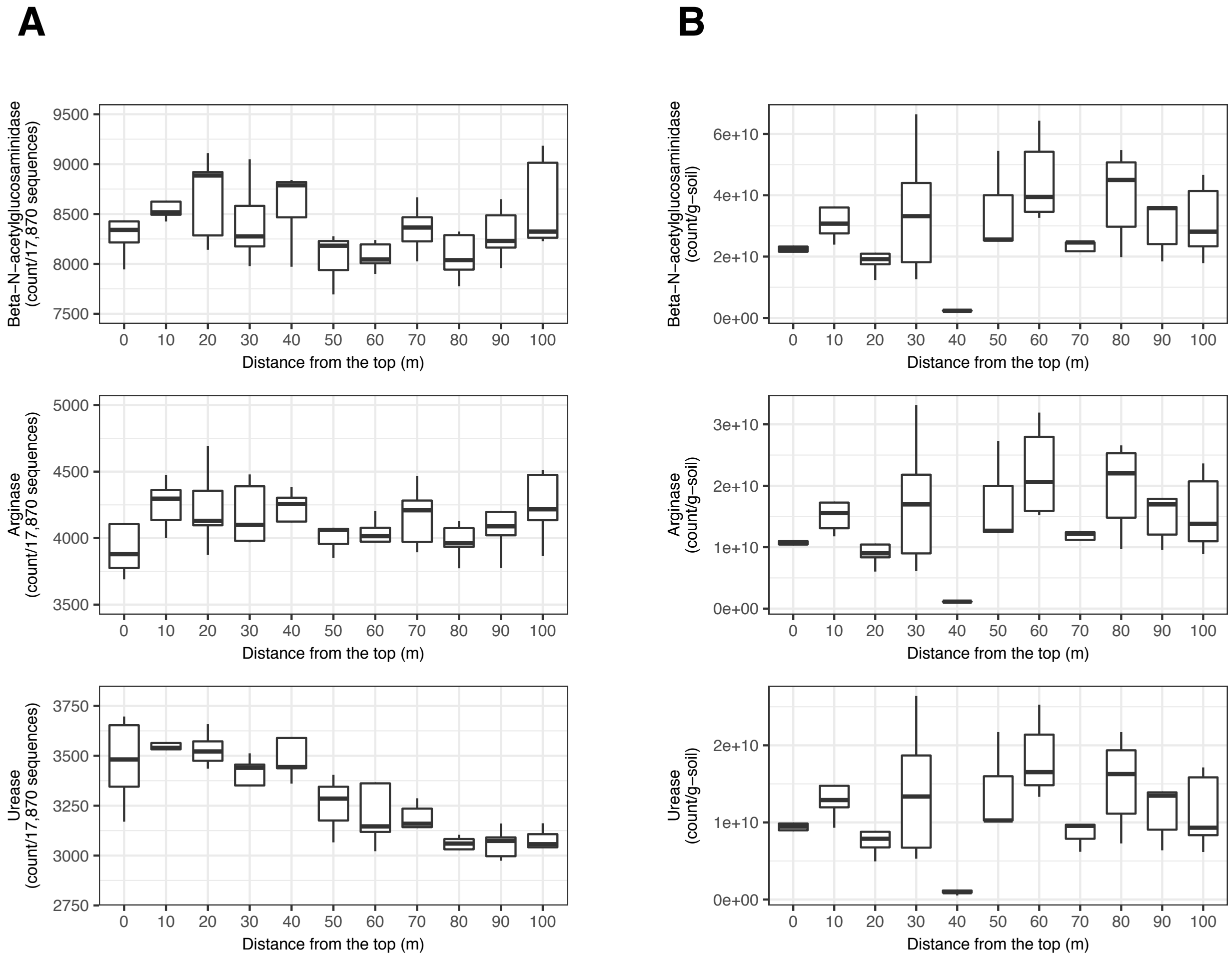

Figure S5 Isobe et al.

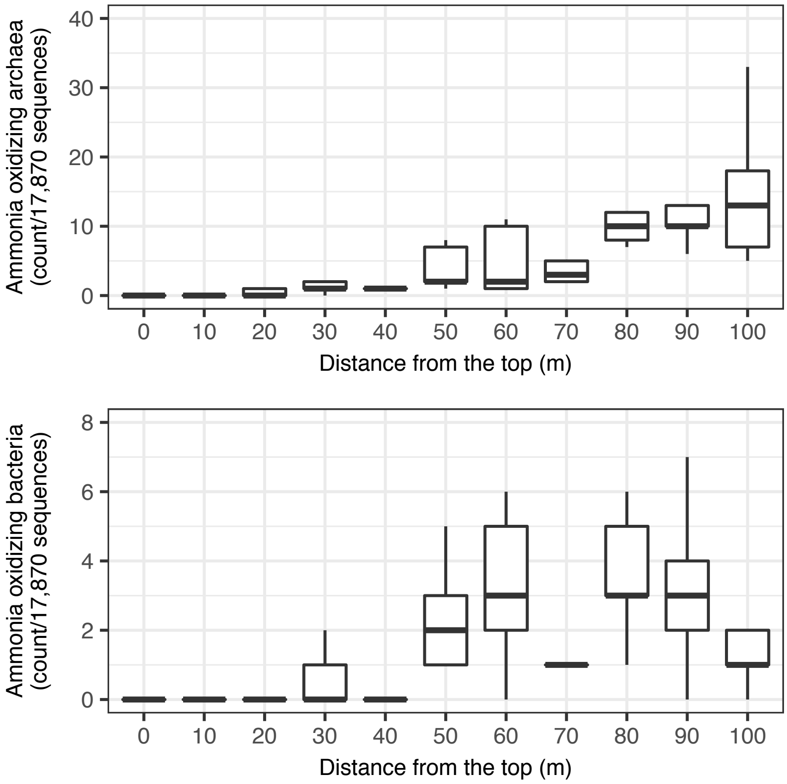

Figure S6 Isobe et al.

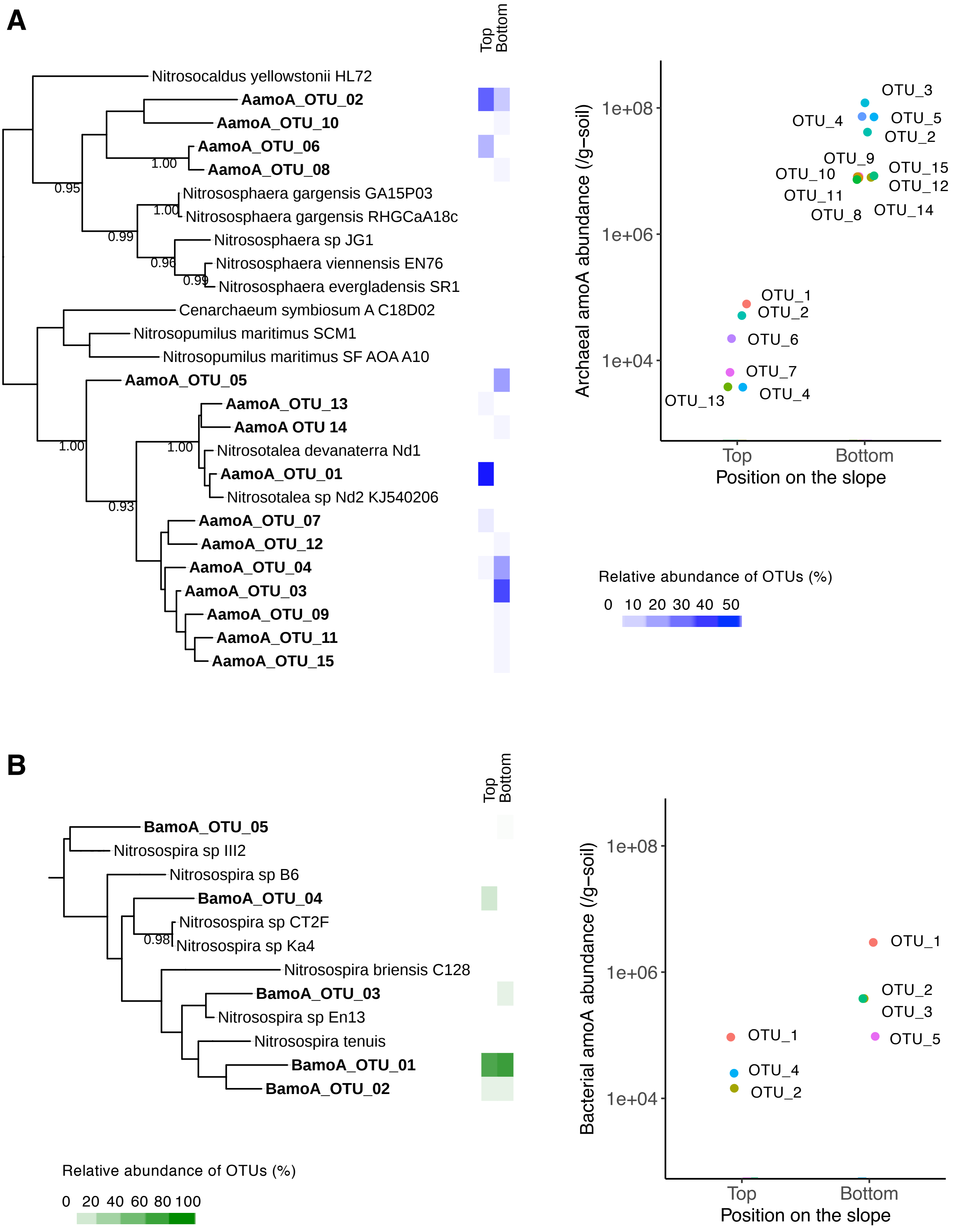

Figure S7 Isobe et al.
